# Structural Studies of Planctomycete *Gemmata obscuriglobus* Support Cell Compartmentalisation in a Bacterium

**DOI:** 10.1371/journal.pone.0091344

**Published:** 2014-03-14

**Authors:** Evgeny Sagulenko, Garry P. Morgan, Richard I. Webb, Benjamin Yee, Kuo-Chang Lee, John A. Fuerst

**Affiliations:** 1 School of Chemistry and Molecular Biosciences, The University of Queensland, Brisbane, Queensland, Australia; 2 Centre for Microscopy and Microanalysis, The University of Queensland, Brisbane, Queensland, Australia; Indian Institute of Science, India

## Abstract

Members of phylum *Planctomycetes* have been proposed to possess atypical cell organisation for the Bacteria, having a structure of sectioned cells consistent with internal compartments surrounded by membranes. Here via electron tomography we confirm the presence of compartments in the planctomycete *Gemmata obscuriglobus* cells. Resulting 3-D models for the most prominent structures, nuclear body and riboplasm, demonstrate their entirely membrane - enclosed nature. Immunogold localization of the FtsK protein also supports the internal organisation of *G.obscuriglobus* cells and their unique mechanism of cell division. We discuss how these new data expand our knowledge on bacterial cell biology and suggest evolutionary consequences of the findings.

## Introduction

Past structural studies [1], [2], [3] have revealed unique cell organisation of the planctomycete bacteria, including the separation of their cytoplasm into compartments via internal membranes [4], [5]. These compartments were designated as ribosome-free *paryphoplasm*, an area between cytoplasmic membrane (CM) and an internal intracytoplasmic membrane (ICM), and the *pirellulosome*, an area surrounded by ICM and containing ribosomes and the DNA of the nucleoid [1]. In the planctomycete *Gemmata obscuriglobus* even a compartment bounded by an envelope, and enclosing the nucleoid DNA, and lying within the pirellulosome, has been suggested [1]. The functions of these structures are obscure due to lack of biochemical and molecular biology studies, so that almost all our current knowledge about the planctomycete cell organisation have been acquired via transmission electron microscopy (TEM) [1], [2], [3]. Previous tomography studies have revealed a complex organisation of the chromosomal DNA [6], but a recent one has not supported intracellular compartmentalisation in *Gemmata obscuriglobus* [7] and instead a tubulovesicular endomembrane system has been proposed [8]. Here we present results of electron tomography performed on whole *Gemmata* cells, which are completely consistent with the originally proposed view [1] of planctomycete cell organisation and its unique compartmentalized nature. Our data on immunogold localisation of the cell division protein FtsK also suggest a new mechanism of cell division, previously not described within Bacteria.

## Materials and Methods

### Cell culture growth and preparation of *Gemmata obscuriglobus* for microscopy

*G. obscuriglobus* was grown on plates containing M1 agar medium [53] incubated aerobically at 28°C for periods of either 3 or 10 days. M1 agar medium plates were prepared as follow: after pouring and allowing agar to set, plates were dried for 1 hour with lids open in a biohazard cabinet. After inoculation by streaking with sterile plastic loop charged with *G. obscuriglobus* grown on the same M1 agar medium, plates were sealed with parafilm before aerobic incubation. After 3 or 10 days of incubation, the cells were collected into an eppendorf tube containing 100 µl of Milli-Q deionized water and examined under phase contrast microscope to check the motility of the cells. 1 µg of GFP was added to the cells, mixed and incubated for 30 min at room temperature, then the cells were examined under fluorescence microscopy (confocal laser scanning) to assess their ability for uptake, as described elsewhere [34]. These standardized methods for medium preparation and culture incubation for 3–10 days were critical for optimal demonstration of GFP uptake [34]. If at least 50% of cells were positive for GFP uptake, another batch of cells from the same dish was collected into 100 µl of Milli-Q deionized water, resuspended, washed with 500 µl of Milli-Q deionized water, then precipitated, and the pellet used for cryofreezing.

### Electron tomography from thick sections

300 nm thick sections were cut using a Leica EM UC6 ultramicrotome. Using these sections, dual-axis tilt-series data were collected at 39000× magnification on an FEI Tecnai F30 operating at 300 kV, over a tilt range of +/−66° at 1° increments for the a-axis and 2° increments for the b-axis, using SerialEM software (The Boulder Lab for 3D Electron Microscopy, USA). Tilt series data of the whole cell were collected at 23000× magnification over a tilt range of +/−66° at 1.5° increments for the a-axis and 3° increment for the b-axis.

### FtsK labelling experiments

The results of preparative work for labelling of sections of G. *obscuriglobus* for TEM via anti-FtsK antibody, including molecular-biological and immunological procedures, antibody testing, and some results of the labelling experiments, can be found in Text S1.

## Results

### Electron tomography reconstructions and 3D models of *G.obscuriglobus* cell compartments

**Figure 1:**
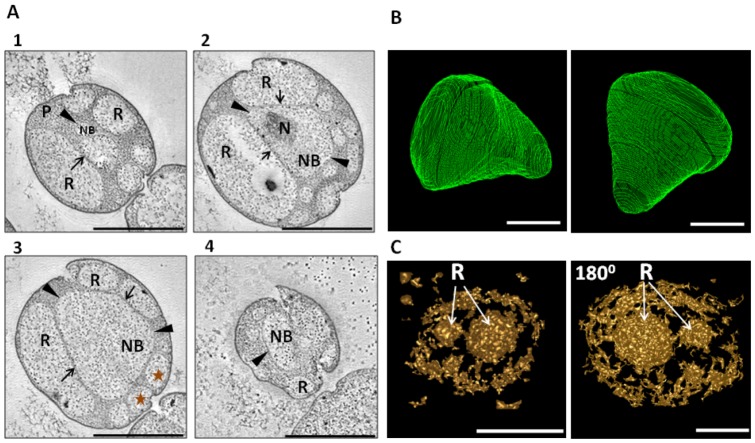
Tomographic reconstruction of a *G. obscuriglobus* cell and 3D models for nuclear envelope and riboplasm. **A**) Transmission electron micrographs of thick-sectioned cryosubstituted (high-pressure frozen) cells showing internal *G. obscuriglobus* compartments. Nuclear body (NB) contains the nucleoid DNA (N), areas of riboplasm (R) contain ribosomes only and no fibrillar nucleoid DNA, and paryphoplasm (P) is ribosome-free. Areas where nuclear body envelope is surrounded by a single membrane are indicated by arrowheads, and where areas of this envelope surrounded by a double membrane by arrows. Numbers 1-4 indicate the order of appearance of a particular image within the tilt-series. Double-membrane nuclear envelope conformation in successive tilt-series is consistent with a continuous surface of double-membrane sheet in these regions, and consistency with membrane continuity is preserved also where single membrane appears to comprise the envelope in certain regions. Stars indicate the riboplasm vesicles used for the 3D model generation (Figure 1C). The whole cell reconstruction can be viewed in Movies S1. Bar, 1 µm. (**B**) and (**C**) 3D models based on the results of electron tomography, from the cell viewed in Figure 1A (see also Movies S1), with extrapolations and manual adjustments every 10 slices. **B**) Nuclear body shown from two different angles. Bar marker, 500 µm. **C**) Riboplasm compartment (R) in the form of vesicles, completely surrounded by membranes, from front and back side (180°) views. Bar markers, 500 µm and 200 µm for the back-side view figure.

Tomographic analyses of three randomly chosen *G.obscuriglobus* cells are demonstrated in the form of movies (Movies S1, S2, S3). For explanation purposes, the slices, or “snapshots” taken from the movies, resulting from tilt-series micrographs of thick sections, are shown in Figures 1A, S1 and S2 in File S1. Movies S1 and Figure 1A demonstrate internal organisation of a whole cell, movie S2 (and Figure S1 in File S1) illustrate organisation of a bud, and movie S3 and Figure S2 in File S1 demonstrate reorganisation of a riboplasm compartment within a *G.obscuriglobus* cell. In all the examined cells paryphoplasm (P) can be seen as the electron-dense ribosome-less area positioned not only along the cell perimeter, but also invaginating inside the cells. Paryphoplasm surrounds ribosome-containing riboplasm (R) and the nuclear body (NB) also containing ribosome-like electron-dense particles. Previously, any ribosome-containing cytoplasm in the pirellulosome region including that surrounding nucleoid has also been called *riboplasm*, but here for convenience in distinguishing *G. obscuriglobus* compartments, this term will be confined to those regions containing ribosomes but not nucleoid. In these particular cells, riboplasm also appears in the form of vesicles, enclosed by a single membrane. The nucleoid DNA (N) occupies the central part of an NB region wholly surrounded by membrane, single in some places and double in others. Closely apposed double membranes seem to occur at the points where riboplasm vesicles border the NB. The NB cytoplasm possesses similar electron density and electron-dense ribosome-like particle distribution to the non-nucleoid-containing riboplasm compartments. 3-D models built upon the results of reconstructions unambiguously confirm the membrane-enclosed nature of both NB (Figure 1B) and riboplasm ‘vesicles’ (Figure 1C). Nucleoid DNA has never been observed in paryphoplasm, and our recent tomographic examinations also confirmed that nucleoid DNA is associated only with the NB compartment [7].

### Visualisation and isolation of *Gemmata obscuriglobus* cell wall

One of the major reasons for recent proposal [7], [9] for the Gram-negative nature of planctomycete cell organisation was inability to recognise the walls of *G. obscuriglobus* cells by electron tomography. However, the walls could be recognised if TEM of whole thin-sectioned cells was applied (Figure S3 in File S1), and they could also be isolated and detected via TEM and negative staining after boiling of cells in 10% SDS (Figure S4 in File S1). Usually, the isolated walls retain not only the cell shape, but also the crateriform structures characteristic of planctomycete walls and surfaces examined via negative staining TEM (Figure S4 in File S1). Thus morphologically the isolated walls resemble the surfaces of the intact cells. This is completely consistent with early studies of the shape-retaining boiling SDS-resistant walls of both *G. obscuriglobus* [10] and other planctomycetes [11], [12].

### Localisation of FtsK protein in *G. obscuriglobus* cells

**Figure 2:**
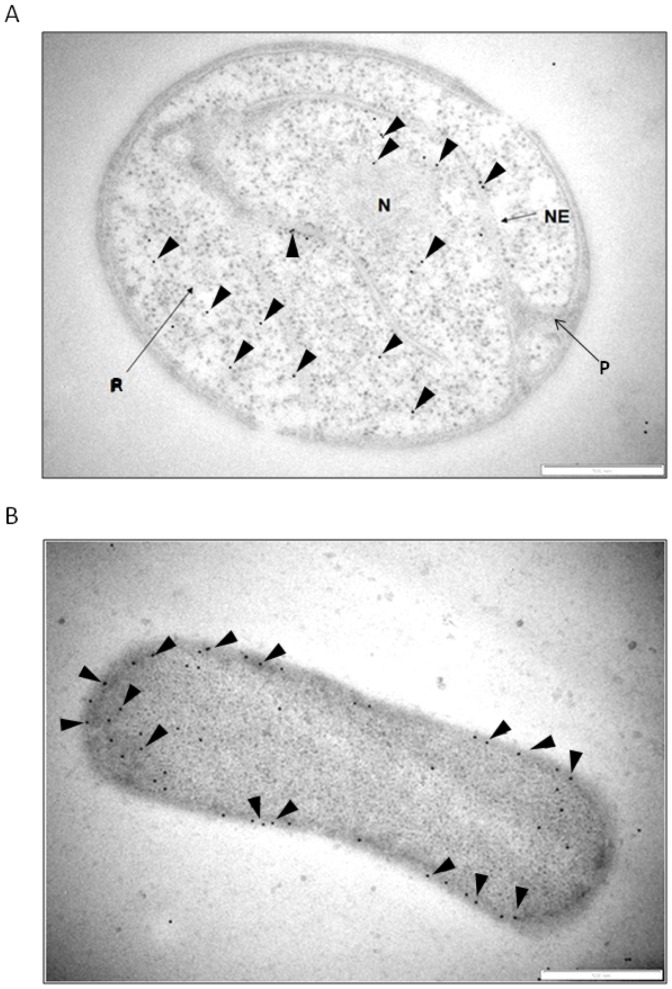
Distribution of the FtsK protein in *G. obscuriglobus* (A) and *E.coli* (B) cells. Immunogold labelling was performed on high-pressure frozen, cryosubstituted, and then thin-sectioned cells. **A**) In *G. obscuriglobus* cells FtsK is localised mostly to the interior of nuclear body (NB) and in riboplasm (R) compartments, but not to paryphoplasm (P). **B**) Instead, in *E.coli* cells FtsK is distributed along the cell periphery (arrowheads). Bar marker, 500 nm. Arrowheads indicate gold particles. Bar marker, 500 nm.

Immunogold labelling of high-pressure frozen and cryosubstituted *G. obscuriglobus* and *E.coli* cells revealed that in *G.obscuriglobus* cells FtsK is localised to nuclear body and riboplasm (Figure 2A), in approximate proportion 2(NB):1(R) (see Text S1 for details), and no significant labelling relative to background was detected in paryphoplasm or on or near its surrounding membrane. As expected, in *E. coli* FtsK was detected along the periphery of the cells (Figure 2B) and also at the sites of cell division (Text S1). Unlike the situation in *E. coli*, *Caulobacter crescentus* and *Bacillus subtilis* [13], [14], [15], [16], [17], where FtsK is a septal ring protein during division, FtsK protein in *G. obscuriglobus* cells was not found attached to a particular membrane or exclusively to the division bud neck where a divisome might be expected, and in some cells the highest labelling pattern was observed over the nucleoid (Text S1).

## Discussion

### Compartmentalisation of planctomycete bacteria

**Figure 3:**
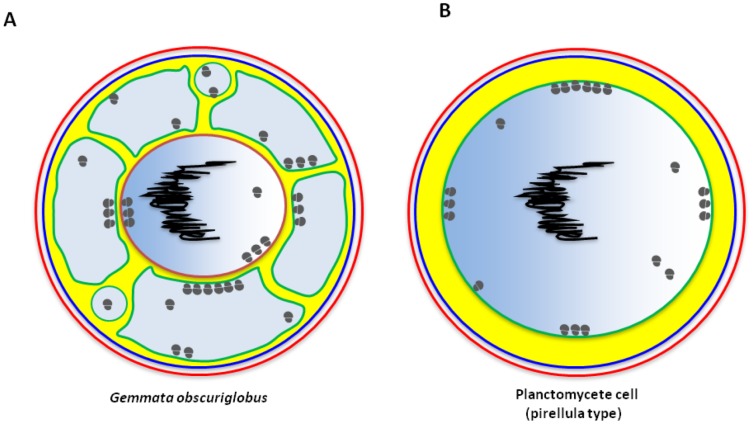
Proposed cell organisations of planctomycetes. **A**) The cell plan for *G.obscuriglobus* proposed in the current publication. This cell plan mostly follows the established view in past publications (as in [4]) including ribosome-less paryphoplasm and intracytoplasmic membrane (ICM), except for presentation of riboplasm, which now appears as multiple vesicles surrounding the nuclear body. Cell wall is indicated in red; cytoplasmic membrane - in dark blue; paryphoplasm - in yellow; riboplasm and nuclear body interior - in light blue; intracytoplasmic membrane - in green; inner nuclear body membrane - in brown; nucleoid DNA - in black; ribosomes – grey circles. **B**) The cell plan for *Pirellula*, which is considered as “simplest” among planctomycetes. A major internal compartment defined by an intracytoplasmic membrane (internal to cytoplasmic membrane bounding the protoplast) encloses a naked nucleoid. Unlike *G. obscuriglobus*, this bacterium thus does not contain a membrane-bounded nuclear body within the major internal pirellulosome compartment. Designation of the structures the same as for (**A**).

In the current work we confirm the enclosed nature of nuclear body and riboplasm compartments in the planctomycete *Gemmata obscuriglobus*, using tomography analyses. In the 3D model for the nuclear body (Figure 1B) every tomographic slice is represented by a green section, and these sections unambiguously show the undisrupted enclosed nature of the nuclear body. The enclosure and separation of the NB contents including nucleoid from other cytoplasm regions found here is consistent with past published structural studies [1], [3], [18]. These results also clarify the structure of the riboplasm compartment. In the cells used for the present tomographic study, this unique type of cytoplasm is represented by a number of vesicles (Figure 1C). These riboplasm vesicles have close relationship with nuclear body, as they exhibit similar electron density, and both possess ribosomes [1], [3] and FtsK protein (see Table S2 in Text S1). In some cells observed in past studies such vesicles may join to form a continuous pirellulosome riboplasm outside a nuclear body surrounded by a double membrane-bounded envelope on all sides [18]. Although such structure could appear as a result of TEM plane of section effects, it may also represent the genuine *in vivo* structure at least at particular cell division stages. A possibility for rearrangements of riboplasm vesicles is demonstrated in Movie S2 and corresponding Figure S2 in File S1. In Figure 3A we schematically show the *G.obscuriglobus* cell plan which appears as a result of the tomography analyses presented here. If we compare it to the plan of the simplest planctomycete cell exemplified by *Pirellula* (Figure 3B), it becomes obvious that the major difference between the plans lies in separation of the riboplasm (pirellulosome) in *G.obscuriglobus* cells into two compartments, nuclear body and the remainder of the riboplasm within the ICM. Membrane invaginations which have been proposed as an alternative of true compartmentalisation [7] may be seen during certain physiological processes such as division of the nucleoid DNA (Figure S5in File S1). In these cells the membrane surrounding nucleoid may be often seen broken at certain points, and even more breaks could be detected in the cells with multiple nucleoids (Figure S6 in File S1). However, substantial rearrangement of membranes in such cells is expected, for purposes of segregation of the nucleoids and transfer of one of them into the bud. Among other cases when membranes may be seen as invaginations inside the core of the cells are: merging/separation of riboplasm vesicles from other riboplasm vesicles (Figure S2 in File S1 and Movie S2) and when riboplasm vesicles merge/separate from the nuclear body (Figures S1 and S7 in File S1, and Movie S2). Presumably, most such membrane reorganisations within a normally physiologically functioning cell are required for maintenance of a certain optimal compartment volume relative to that of the cell. For instance, in the bud cell shown in Movie S2 and Figure S1 in File S1 we apparently see stages in formation of a large riboplasm “vesicle” via separation from just transferred nuclear body. This proposition may be supported by the fact that the volume of the nuclear body in a *G.obscuriglobus* mother cell is usually considerably lower than in the bud cell (Movie S2 and Figure S1 in File S1). The smaller volume of the nuclear body and riboplasm vesicles may facilitate such biochemical processes in the cells as protein synthesis and DNA replication via molecular crowding. A similar phenomenon may occur in protist cells when vesicles (called “transport vesicles”) are formed in a stage of replication of the giant Mimivirus infecting amoeba cells. Those vesicles appear to be formed from the nuclear envelope or rough endoplasmic reticulum membrane closely associated with nuclear membranes in such amoebae [19]. Within the Archaea, *Ignicoccus* cells can display vesicle-like structures between an inner membrane and an ‘outer cellular membrane’ [20], [21] with features such as ATP synthase [22] suggesting characteristics of a cytoplasmic membrane. *Ignicoccus* structure may thus be usefully compared with that of planctomycetes, and may conceivably also be compartmentalized.

### The ‘Gram-negative’ controversy and periplasm in planctomycetes

One of the difficult questions posed by *G.obscuriglobus* and other planctomycetes is the location of periplasmic space in these bacteria. In bacteria such as *E.coli* with a classical Gram-negative cell wall structure, the periplasmic space containing a gel-like ‘periplasm’ lies between an outer wall membrane (the ‘outer membrane’ of the wall in *E.coli*) and the cytoplasmic membrane enclosing the cytoplasm [23], [24]. A thin peptidoglycan layer essential for the structural integrity of such Gram-negative walls, preventing cell lysis via osmotic effects in dilute media, lies within this periplasmic space. In contrast, planctomycetes including *G. obscuriglobus* possess a wall composed mostly of protein as the structurally rigid layer of the wall, without any detectable peptidoglycan [10], [12], and there is no associated membrane external to this layer – this protein layer lies over and closely apposed to the cytoplasmic membrane. It should be noted that planctomycetes give a Gram-negative reaction in classical Gram staining procedures, but this is not a reflection of affinity with a Gram-negative wall of the *E.coli* type – they have completely distinct wall structure. Thus planctomycetes cannot be referred to as typical Gram-negative bacteria, or their cell structure misinterpreted in the light of this [9], [25], [26], [27].

Hypothetically, in planctomycetes the area between proteinaceous cell wall and cytoplasmic membrane could serve as periplasmic space. This makes the *G.obscuriglobus* cell surface similar to those of some Archaea [28] and to those of Gram-positive bacteria where a periplasm appears to exist despite the absence of a Gram-negative wall outer membrane [29], as well as perhaps to another well-studied compartmentalized planctomycete, *Kuenenia suttgartiensis*, where an S-layer protein wall overlies an outermost membrane [30]. Such Gram-negative bacteria as *Caulobacter crescentus* also have an outermost proteinaceous layer [31] but it is attached to a lipopolysaccharide-containing outer membrane and in addition they possess a typical peptidoglycan layer between the outer and inner membranes, and conventional bacterial cell division system [32]. The proposal [7], [9] that paryphoplasm of planctomycetes is actually periplasm so that the cytoplasmic membrane is reinterpreted as outer membrane [33] meets a number of serious difficulties. Paryphoplasm possesses abundant membranes and small vesicles (about 50 nm in diameter), functions of which are largely unknown, but at least some of which seem to function in endocytosis-like protein uptake [34]. In contrast to periplasm, which appears to be a uniform gel-like layer in Gram-negative bacteria [35], there is an apparent spatial separation of several processes in the paryphoplasm. The presence of the vesicles, observed via electron microscopy of sectioned cells, is also implied by the observed degradation of endocytosis-acquired proteins [34], [36] and this makes paryphoplasm more similar to cytoplasm of the eukaryotic cells, rather than to periplasm of bacteria. This is consistent with the finding of RNA in paryphoplasm of planctomycetes as well as in riboplasm and nuclear body, where expected [1], [37]. MC-like protein, which is considered to be an analog of the eukaryotic clathrin, is also localised to paryphoplasm, again consistent with its cytoplasmic rather than periplasmic nature [38]. In any case the proposal of Gram-negativity of the planctomycete wall and consequent rejection of the paryphoplasm depends at present solely on genomic evidence suggesting presence of such genes as those for lipid A synthesis and outer membrane proteins [33], but the location of relevant gene products in any planctomycete has not yet been determined. So such bioinformatic analysis can have no definite implications for interpretation of cell structure and can easily lead to misinterpretation without further localization evidence.

### New mode of planctomycete cell division

**Figure 4:**
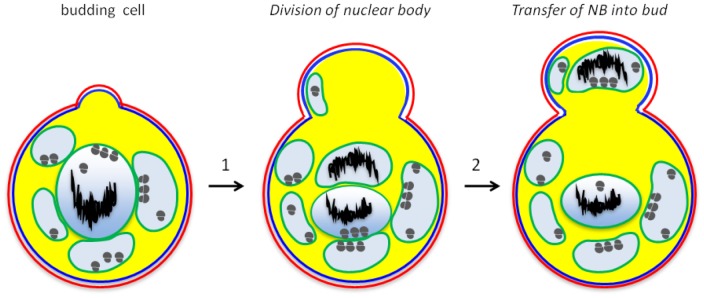
A model for mechanism of cell division of *G. obscuriglobus* cells. Step 1, the bud appears as a hump on the surface of a cell (Figures S5 and S6 in File S1). The nuclear body is divided, before or during the formation of a bud, forming two fully enveloped structures, as shown in step 2. Finally, one of the nuclear bodies migrates into a newly formed cell (step 3). Other riboplasm vesicles not containing nucleoid DNA are also transferred into the newly formed cell (Figure S6B in File S1). Cell wall is indicated in red; plasma membrane – blue; ICM – green; paryphoplasm – yellow; riboplasm – light blue; nucleoid – black; ribosomes – grey circles.

Our current results also allowed us to clarify some important aspects of *G.obscuriglobus* cell division. One argument against compartmentalisation of planctomycetes is that a complex membrane system in *G. obscuriglobus* would experience major problems in membrane separation during the cell division process [7]. This suggestion would only apply however in the case where membranes are chaotically distributed within the cell interior. The current work instead suggests that *G. obscuriglobus* cells possess a unique cell division mechanism not involving a divisome depending on such septal proteins as FtsZ and FtsK, but involving chromosome segregation via pre-separation of chromosomes in multiple mother cell nuclear bodies. The major players in division of both Gram-negative and Gram-positive bacteria are Fts proteins including FtsZ and FtsK among many others, responsible for formation of the Z-ring and divisome at the division site. If the planctomycete cell organisation resembles that of Gram-negative bacteria [7], [9], [33], [39], localisation of FtsK should resemble that in other Gram-negative bacteria, and be consistent with an FtsZ-centred divisome. However, in addition to absence of peptidoglycan, absence of FtsZ from all planctomycete genomes [40] is inconsistent with a Gram-negative division mode but suggests existence of alternative modes [41]. Planctomycetes do possess the bacterial division protein FtsK, which was found in the internal milieu of riboplasm and nuclear body (Figure 3 and Text S1). It also was found bound to nucleoid, not to the ICM membrane, even though membrane binding and especially cytoplasmic membrane binding would be expected in the case of a Gram-negative model of FtsK function, and even though FtsK in *G. obscuriglobus* does possess predicted membrane-binding regions (Text S1). Thus, the function of FtsK may be restricted in *G.obscuriglobus* only to chromosome partitioning, and another, yet unidentified system may be responsible for segregation of the nuclear body membrane [42]. FtsK in *E.coli* appears to be localized to membrane and after forming hexameric assemblies, is recruited to septal membrane upon division initiation [42]. If the membrane domain of FtsK is deleted, the protein can still function in chromosome segregation via the opening between mother and daughter cell [43], but is presumably still part of an FtsZ-centred divisome. Even in ZipA-depleted *E. coli* mutants, FtsK is localized to cytoplasmic membrane and not nucleoid or cytoplasm [44]. Association of FtsK with the nucleoid in *G.obscuriglobus* as found here may be similar to the association of plasmid partitioning proteins with nucleoid in *E.coli* [45]. We depict here a proposed model for the cell division process (Figure 4) which does not involve any difficulties during budding and DNA segregation. According to the model, the mother cell about to divide just requires segregating the nuclear body compartment into two, and each of them should contain nucleoid. Then, one of the nuclear bodies is transferred into a newly produced bud, as can be deduced from Movie S2 and Figure S1 in File S1.

Thus, true compartmentalisation involving membrane-bounded compartments internal to the cytoplasm is present in the planctomycete *Gemmata obscuriglobus* - there is definitely a growth state (in cells after 3–5days of incubation on M1-agar medium, which corresponds to young actively dividing and endocytosis-capable culture) when cells have a clearly membrane-separated compartment containing the nucleoid DNA, bounded at different points in at least a single membrane and in significant major portions by two closely apposed membranes. Tomographic reconstructions do not suggest any points where the nucleoplasm is connected with other cytoplasm – at least a single membrane separates them. As judged from this and other studies of *G. obscuriglobus*, the ribosome-less region characteristic of all planctomycete cell plans, the paryphoplasm, cannot be considered as periplasm due to its uniqueness in structure and function.

### Implications for cell biology and evolution

The results here are consistent with other arguments for the exceptional nature of planctomycetes and related bacteria and their importance for understanding evolution [46]. The confirmation of membrane-bounded compartmentalization of the cytoplasm in a bacterium strengthens the view that planctomycete structure may form a model for understanding how eukaryote compartmentalization evolved, including the nucleus and its envelope [47], [48], [49]. It is entirely consistent with the existence of other remarkable similarities with eukaryote cell biology in this species such as endocytosis-like protein uptake [34]. Demonstration of evolutionary homology will await molecular genomic evidence though indications of some homologs may already exist e.g. MC (membrane-coat) protein homologs [38] and endocytosis-related proteins [50]. However, to understand any underlying deep similarity in structure of the nuclear body of *G. obscuriglobus* with the nucleus of eukaryotes, it may be useful to compare the topology of the structure of the eukaryote nuclear envelope with that of *G. obscuriglobus*. Both have envelopes which have major regions with two closely apposed membranes, and in both cases there are short regions where the outer membrane extends to form connections with other intracellular membranes. In eukaryotes such outer nuclear membrane connects the nuclear envelope with the ER, and in *G. obscuriglobus* the outer of the apposed nuclear membranes in ‘double’ regions connects with the ICM. This means that in both cases, membrane completely surrounds the nuclear compartment, but not necessarily at all points via a double membrane or even by closed membrane sheets. Additional complications to topology arise from nuclear pore complexes, the likely possibility of which is still under investigation in *G. obscuriglobus*. Regardless of evolutionary homology with eukaryotes, the *G. obscuriglobus* compartments will form a useful model for how internal eukaryote compartments could have arisen in a proto-eukaryote by endogenous mechanisms [48] independent of any later endosymbiosis such as the introduction of mitochondria. Major features of eukaryote cell biology molecular mechanisms were present in the last eukaryotic common ancestor, including features of nuclear envelope [51], [52]. Exploration of molecular cell biology underlying and perhaps ancestral to these similarities may yield new mechanisms for complex membrane development in cells, if not the deepest ancestral forms of homolog with eukaryote complexity. Evolutionary models for how such homology came about [47] can then be tested.

In summary, the origin of the eukaryote endomembrane system and membrane-bounded nucleus are major unsolved problems in evolutionary biology. Yet there are no clear experimental models for testing the two major hypotheses for such origin - an endosymbiotic engulfment versus an endogenous membrane invagination in a protoeukaryote. However, using the model organism *Gemmata obscuriglobus* we confirm here that planctomycetes do represent an example of bacteria possessing a structural analogue of the nucleus of eukaryotes, with respect to its compartmentalized separation by membranes from the rest of the cell. They may thus constitute useful models for future experimental test at a molecular level for how endogenous membrane invagination could give rise to endomembrane systems and the nucleus.

## Supporting Information

File S1 **Figures S1–S7.** Figure S1. Tomographic reconstruction of a *G. obscuriglobus* bud. Transmission electron micrographs of thick-sectioned cryosubstituted (high-pressure frozen) cells showing internal *G. obscuriglobus* compartments of a bud. Possible formation of a riboplasm vesicle from a nuclear body can be seen. The processing was done as for the cell shown in Figure 1. Numbers 1-4 indicate the order of appearance of a particular image within the micrographs. Arrowheads in image 2 indicate invaginations of the nuclear body membrane. Inset in image 3 indicates the point of budding at the final stage, where interconnection of the cells still occurs. Image 3 shows that at the more distant point, compared to image 2, nuclear body and riboplasm are fully separated by membranes. Arrowheads in 1, 3 and 4 indicate single nuclear body membrane, and arrows indicate double membrane. Other designations: nuclear body (NB), nucleoid DNA (N), riboplasm (R), paryphoplasm (P). The whole cell reconstruction can be viewed in Movie S2. Bar, 500 nm. Figure S2. Partial tomographic reconstruction of a *G. obscuriglobus* cell. Transmission electron micrographs of thick-sectioned cryosubstituted (high-pressure frozen) cells consistent with the proposal that riboplasm vesicles may rearrange (fuse or separate from each other). Arrowheads indicate membrane invaginations inside a riboplasm vesicle (R) either representing a process leading to breakage of the vesicle onto two separate units or a joining of two pre-existing vesicles. Within the cells are seen: nuclear body (NB) with nucleoid DNA (N), riboplasm (R) and paryphoplasm (P). Numbers 1-3 indicate the order of appearance of a particular image within the tilt-series. The partial cell reconstruction can be viewed in Movie S2. Bar, 200 nm. Figure S3. Internal compartments in *G. obscuriglobus* cells. Whole cells were thin-sectioned after cryosubstitution processing and resin embedding, then examined under TEM. The interior of a cell is compartmentalized by membranes into nuclear body (NB) containing the nucleoid DNA (N), areas of riboplasm (R) containing ribosomes only and no fibrillar nucleoid DNA, and ribosome-free paryphoplasm (P). Bar, 500 nm. The inset enlargement of the boxed area shows cell wall (black arrowheads), which appears as an outermost thin layer. Cytoplasmic membrane is indicated by white arrows, and intracytoplasmic membrane by white arrowheads. P –paryphoplasm; R – riboplasm; NB – nuclear body, containing nucleoid DNA (N). Bar, 50 nm. Figure S4. Cell walls of *G.obscuriglobus*. Cell walls of *G.obscuriglobus* isolated by boiling in 10% SDS. A) A clump of bacterial walls viewed via TEM after negative staining with uranyl acetate, which are relatively electron-transparent and retain the round shape of intact untreated cells. The transparency indicates that the interior is lacking the intracellular material. Bar, 5 µm. B) Magnified image of negatively stained cell wall shows characteristic crateriform structures on the surface (arrowheads). Bar, 200 nm. C) The isolated cell walls as viewed after cryosubstitution and thin-sectioning. Bar, 1 µm. D) Inset from (C) showing a thin cell wall layer (arrow) with crateriform structures (arrowheads). Bar, 100 nm. E) TEM image of a *G.obscuriglobus* wall, isolated by boiling in 10% SDS. A single cell wall layer is indicated by black arrows, and a crateriform structure by a white arrow. Bar, 50 nm. Figure S5. Membrane rearrangements in a budding *G.obscuriglobus* cell. TEM images of a non-budding cell (A), where paryphoplasm (P), riboplasm (R), and nuclear body (NB) containing nucleoid (N), are clearly seen, and a budding cell (B), where some of the internal membranes are not interconnected (black arrowheads). A bud in process of formation (white arrowhead) and two nucleoids (N) are indicated. Bar, 500 nm. Figure S6. Multiple nucleoids in *G. obscuriglobus* cells. Whole cells were thin-sectioned after cryosubstitution processing and resin embedding, then examined under TEM. The interior of the cells is separated by membranes (arrowheads) which surround nucleoids (N). A) A cell which contains four nucleoids, two of which (N1 and N2) are fully enclosed by membranes and separated from the other two nucleoids (N3 and N4). Bar, 500 nm. B) A budding cell which contains four nucleoids, two of them (N1 and N2) fully enclosed by membranes and separated from the other two nucleoids (N3 and N4). The bud is indicated by a white arrowhead. Bar, 500 nm. C) A cell which is interpreted as having just finished budding, containing three nucleoids, two of them (N1 and N2) clearly fully surrounded by membranes. The former bud is indicated by white arrow. Bar, 2 µm. Figure S7. Membrane rearrangements in a *G. obscuriglobus* cell. TEM images of thin section of a whole cryosubstituted *G.obscuriglobus* cell which is apparently in a state of budding, since three nucleoids (N) within the nuclear body (NB) are seen. Paryphoplasm (P) is seen as dark areas, while riboplasm (R) appears as more transparent areas. The star indicates a riboplasm “vesicle”, which appears as if merging with or separating from the nuclear body. Arrowheads indicate the places where membranes are expected. Bar, 500 nm.
(PPTX)

Text S1 **FtsK localization within** ***Gemmata obscuriglobus*** **cells: preparation of antibody and immunogold staining.**
(DOCX)

Movie S1 **Tomographic reconstruction of a** ***G. obscuriglobus*** **cell.** Detailed description of the preparation of the cell appearing in the movie can be found in the legend for Figure 1, which was generated from Movie S1.
(MOV)

Movie S2 **Tomographic reconstruction of a** ***G. obscuriglobus*** **bud.** Detailed description of preparation of the cell appearing in the movie can be found in the legend for Figure S2 in File S1, which was generated from Movie S3.
(WMV)

Movie S3 **Partial tomographic reconstruction of a** ***G. obscuriglobus*** **cell showing membrane reorganisations which involve riboplasm vesicles.** Detailed description of the preparation of the cell appearing in the movie can be found in the legend for Figure S2 in File S1, which was generated from Movie S3.
(MOV)
