# Supplementary material for: Structural Studies of Planctomycete Gemmata obscuriglobus Support Cell Compartmentalisation in a Bacterium
Source: PLoS One. 2014 Mar 14;9(3):e91344. doi: 10.1371/journal.pone.0091344 (PMC3954628; doi:10.1371/journal.pone.0091344)
Supplement: Text S1 — FtsK localization within Gemmata obscuriglobus cells: preparation of antibody and immunogold staining. (DOCX) [file pone.0091344.s002.docx]

**Text S1**

**FtsK localization within *Gemmata obscuriglobus* cells: preparation of antibody and immunogold staining**

### Materials and Methods

### Retrieving *ftsK* gene sequence from the draft *G. obscuriglobus* genome

FtsK protein sequences of 16 bacterial strains were retrieved from the ExPASy (**Ex**pert **P**rotein **A**nalysis **Sy**stem) Proteomics Server. These strains included *Agrobacterium tumefaciens*, *Bacillus subtilis*, *Chlamydia pneumonia*, *Dehalococcoides sp*., *Escherichia coli*, *Leptospira interrogans*, *Lactobacillus sakei*, *Mycobacterium leprae*, *Pseudomonas syringae*, *Rickettsia prowazekii*, *Staphylococcus aureus*, *Streptomyces avermitilis*, *Salmonella typhi*, *Treponema pallidum*, *Vibrio parahaemolyticus* and *Xanthomonas axonopodis*. The *E. coli* *fts*K amino acid sequence was used to blast against the *Gemmata obscuriglobus* draft genome in TIGR (**The Institute for Genomic Research**) databases, the contig containing a possible *fts*K gene sequence was retrieved, and the open reading frame (ORF) was detected using ORF Finder in NCBI (National Center for Biotechnology Information) databases (<http://www.ncbi.nlm.nih.gov/gorf/>). The retrieved ORF was confirmed to be an FtsK homolog by blasting against the NCBI protein database via Basic BLAST blastp (<http://blast.ncbi.nlm.nih.gov/Blast.cgi?PROGRAM=blastp&BLAST_PROGRAMS=blastp&PAGE_TYPE=BlastSearch&SHOW_DEFAULTS=on&LINK_LOC=blasthome>).

### FtsK protein sequence alignment

*G. obscuriglobus* Ftsk protein sequence was aligned with 16 FtsK protein sequences of other bacterial strains using Clustalx, and searched for any conserved regionss. GeneDoc ver. 2.6.002 was used for final editing to produce the final aligned sequences.

### Cloning of *fts*K gene from *Gemmata obscuriglobus*

A pGEX-KG expression vector was kindly provided by Drs. Thierry Lonhienne and Bernie Carroll, The University of Queensland. PCR product was first digested with *Nco*I and *Hind*III restriction enzymes overnight and ligated with Promega 2× rapid ligation buffer at 37°C for 6 hours. Plasmids containing inserts (pGEX-KG_FtsKc) were transformed into *E. coli* JM109 competent cells. Plasmids containing inserts were extracted using a Roche High Pure Plasmid Isolation Kit and transformed into *E. coli* Bl21 competent cells.

### Recombinant protein extraction

1 litre of the IPTG-induced *E. coli* culture expressing the protein of interest was centrifuged at 6,000 rpm for 15 minutes at 4°C in centrifuge bottles using a Beckman J2-MI centrifuge with Type JA-14 rotor. From now on, all steps were performed at 4°C until specified. Supernatant was removed and the weight of the pellet was determined (approximately 4 g). For each gram (wet weight) of *E. coli* pellet, 3 ml of cell lysis buffer was added to resuspend the pellet. For each gram of *E. coli*, 4 µl of 100 mM PMSF was added and then 80 µl of 10 mg/ml lysozyme. The suspension was stirred for 20 minutes with a magnetic stirrer. While stirring was continued, 4 mg of deoxycholic acid per gram of *E. coli* was added to the mixture. The suspension was then stored at 37°C and stirred occasionally with a sterile glass rod. When the lysate became viscous, 20 µl of 1 mg/ml DNase I was added per gram of *E. coli*. The lysate was stored at RT until it was no longer viscous (approx. 30 minutes). The cell lysate was centrifuged at 14,000 rpm for 15 minutes at 4°C using a Hettich EBA-12 benchtop centrifuge and the supernatant was filter-sterilized and stored at 4°C.

### Affinity chromatography purification

50% slurry of Glutathione Sepharose 4B (Amersham Biosciences) was added to the affinity column to give a final 2 ml bed volume. The medium was equilibrated with 1× PBS (5 bed volume) and drained. GST-FtsKc fusion protein extract was added to the column and incubated for 30 min at RT. The column was drained, flow-through was collected and added back to the column and incubated for another 30 min to maximise the protein capture. The column was drained and the medium was washed with 10 bed volumes of 1× PBS. The wash was repeated twice more for a total of three washes. After the unbound proteins had been washed and drained, the column outlet was closed. Elution buffer was added to the column (1 ml per 1 ml bed volume) and incubated at RT for 10 min. GST-FtsKc fusion protein was then eluted and collected. The elution step was repeated twice more and all three eluted fractions collected. The medium was then regenerated and kept at 4°C for the next purification.

### Antibody purification using Protein-G sepharose

Anti-FtsK antibody was purified from rabbit terminal bleed using Protein-G sepharose. 700 µl of filter-sterilized terminal bleed was diluted with 200 µl of 1× PBS. 50 µl of Protein-G sepharose was dispensed into a 1.5 ml eppendorf tube and re-equilibrated with 500 µl of 1× PBS twice. The tube was centrifuged at 1,800 g for 1 min and supernatant was discarded. Pelleted Protein-G-sepharose was resuspended with 900 µl serum mixture and incubated for 20 min at 4°C. The tube was centrifuged at 650 g for 3 min and supernatant discarded. 20-min incubation with a fresh 1 ml serum mixture was repeated 6 more times. The tube was centrifuged at 650 g for 3 min and the supernatant was discarded. 3 washes of 1× PBS were then performed. The tube was centrifuged at 1,800 g for 1 min and the supernatant was discarded. Antibody was eluted by adding 150µl of 0.2M glycine/HCl, pH2.5 and centrifuged at 1,800 g for 1 min. Supernatant was transferred to a tube containing 50 µl of neutralization buffer followed by addition of 100 µl 50% glycerol and stored at -20°C. Protein-G sepharose was re-equilibrated with neutralization buffer twice (each 900 µl) followed by 3 washes with 1× PBS and one wash with 350 µl of 20% ethanol. Protein-G sepharose was resuspended with 75 µl of 20% ethanol and the beads stored at 4°C which were then ready for another purification process.

### Transmission electron microscopy

All high-pressure frozen and cryosubstituted sections were viewed using a JEOL 1010 transmission electron microscope operated at 80 kV. Images were captured using iTEM 5.0, universal TEM image platform software. The resulting files were annotated for final image production using Photoshop CS.

#### High-pressure freezing

*Gemmata obscuriglobus* examined using transmission electron microscopy was high-pressure frozen with liquid nitrogen using a Leica EMPACT 2 high-pressure freezer. The frozen samples were stored in a 2 ml tube containing liquid nitrogen before cryosubstitution. *E. coli* was also frozen and processed which was later used as positive control in the immunogold labelling experiments.

#### Cryosubstitution

Each Leica carrier containing frozen sample was transferred to an eppendorf tube containing 0.2% uranyl acetate and 5% water in acetone and cryosubstituted in Leica AFS. The sample was warmed from -140°C to -85°C over 2 hr (rate 27.5°C/hr); stored at -85°C for 50hrs; and finally warmed -85°C to -50°C over 11.7 hrs (3°C/hr). The high-pressure frozen and cryo-substituted samples were infiltrated into Lowicryl HM20 resin also in Leica AFS. At -50°C samples were first incubated with Lowicryl HM20 diluted in acetone (1:1) for 1 hr followed by 1 hr incubation with Lowicryl HM20 diluted in acetone (2:1 respectively. Samples were finally incubated twice with 100% Lowicryl HM20 resin solution initially for 4 hrs and then for 8 hrs. Samples were then polymerised in Lowicryl HM20 for 48 hrs at -50°C followed by 48 hrs at 20°C. The resin block was thin-sectioned using the Leica Ultracut Ultramicrotome UC61 to obtain thin-sections with 70-90 nm thickness. Thin-sections were placed onto a pioloform-coated and carbon-coated copper grid for later use in immunogold labelling.

#### Immunogold labelling using anti-FtsK antibody

Immunogold labelling was always performed using the Pelco BioWave microwave oven to obtain a result in less time compared to a conventional technique performed on the bench. Carbon-pioloform-coated copper grids containing thin-sectioned specimens were floated onto drops of Blocking solution (1× PBS, 20 mM glycine, 0.2% fish skin gelatine and 0.2% BSA) on a sheet of parafilm with microwave on for 1 min at 150w. Grids were then transferred onto 8 µl of rabbit polyclonal anti-FtsK antibody produced in this study (diluted 1:10 in Blocking solution) for 2 min on, 2 min off and 2 min on in the microwave at 150w. Grids were washed with Blocking solution 3 times each for 1 min using the microwave on at 150w. Grids were then placed onto 8 µl of protein A-10nm gold (Cell Microscopy Centre, Department of Cell Biology, University Medical Centre Utrect, Netherlands) diluted 1:75 in Block solution for 2 min on, 2 min off and 2 min on in the microwave at 150w. The grids were then washed three times with 1× PBS followed by 3 washes with Milli-Q grade deionised water and each microwaved for 1 min at 150w. The grids were then dried on Whatman grade 1 filter paper and kept in a storage grid box for TEM examination.

### Quantitative analysis of FtsK immunogold labelling

Transmission electron micrographs of gold-labelled *G. obscuriglobus* cells were viewed using iTEM 5.0, universal TEM image platform software. The area of different compartments within the cell was measured and the density of gold particles per unit area was determined. The paryphoplasm compartment was defined as the region between the ICM and the cytoplasmic membrane; the pirellulosome compartment was defined as the region enclosed by the ICM within the cell including the double-membrane bounded nuclear body; the nucleoid DNA was defined as the dense fibrillar nucleoid within the nuclear body; nucleoid envelope was defined as the double-membrane surrounding the nucleoid DNA. Background labelling was also determined by measuring the background area defined as the total area minus the area of each cell present in the micrograph and measuring the density of gold particles per unit area of that background so defined.

### 2. Characterisation of *G. obscuriglobus* FtsK protein

*G. obscuriglobus* *fts*K gene sequence (2433 bases) was retrieved from the draft genome at TIGR database via Blast search using *E. coli* *fts*K protein sequence as the query. The open reading frame of the protein was detected using NCBI ORF finder. The protein sequence was examined using two computational algorithms TMHMM Server, v.2.0 (available free at <http://www.cbs.dtu.dk/services/TMHMM-2.0/>) and TopPred (available free at <http://mobyle.pasteur.fr/cgi-bin/portal.py?form=toppred>) for predicting transmembrane helices which is a common feature of FtsK at the N-terminus domain as in *E. coli* and *B. subtil*. Figure S8 shows the hydrophobicity profile of *G. obscuriglobus* FtsK and from this it is predicted to possess 4 possible transmembrane helices at the N-terminus region and a large hydrophilic C-terminal domain.


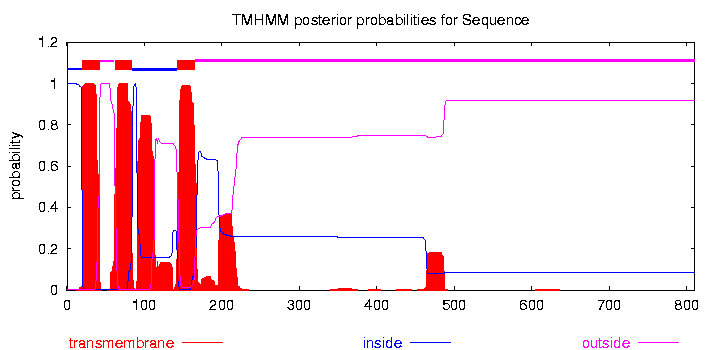

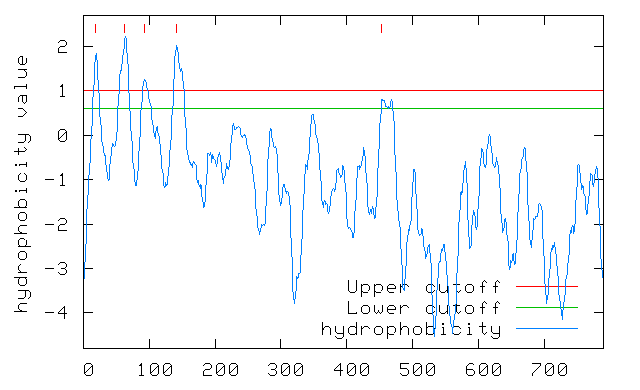


**Figure S8.** Hydrophobicity profiles of *G. obscuriglobus* FtsK protein sequence showing 4 predicted transmembrane helices at the N-terminal domain. Top – prediction from TMHMM, Bottom – prediction from TopPred, both with similar profile.

*G. obscuriglobus* FtsK protein amino acid sequence was used as a query to search against the protein database at NCBI website using Specialized BLAST to search conserved domains (<http://www.ncbi.nlm.nih.gov/Structure/cdd/wrpsb.cgi>). Figure S9 shows that *G. obscuriglobus* FtsK possesses two conserved domains at the C-terminal region including a P-loop NTPase superfamily domain and an FtsK gamma domain.


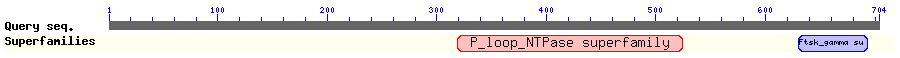


**Figure S9.** Conserved domains possessed by *G. obscuriglobus* FtsK include P-loop NTPase superfamily and FtsK gamma domain using specialized BLAST.

### FtsK sequence alignment

*G. obscuriglobus* FtsK protein sequence was aligned with 16 other reference FtsK sequences using clustalx and edited using GeneDoc. According to the alignment, the N-terminal domain and central linker domain of FtsK are variable among bacterial species. The C-terminus region on the other hand is quite conserved (Figure S10) which is consistent with the result of the Specialized Conserved Domain Blast (Figure S9). Two active motifs, Walker A and Walker B are also found to be highly conserved among the bacterial species at the C-terminal domain. Sequences encoding these motifs are highlighted in a red box. Walker A and Walker B domains form part of the region defining the P-loop NTPase superfamily domain recognised by conserved domain blast. *G. obscuriglobus* FtsK also possess a conserved gamma domain that is DNA binding and interacts with KOPS.


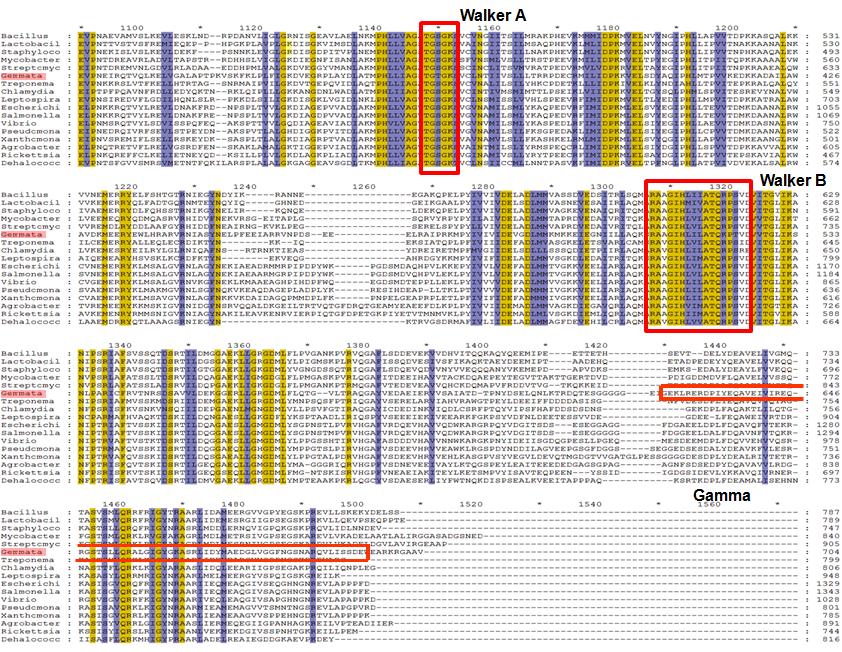


**Figure S10**. FtsK protein sequence alignment of 17 bacterial strains including G. obscuriglobus showing the conserved C-terminal region. Red boxes highlight Walker A, Walker B and DNA-binding FtsK gamma subdomains. Pink region highlights the position of *G. obscuriglobus*.

1. **Preparation of anti- *G. obscuriglobus* FtsK antibody**

### Cloning of ftsK gene from *Gemmata obscuriglobus*

The partial C-terminus region of *G. obscuriglobus fts*K gene was PCR-amplified. Sequences for restriction enzymes NcoI and HindIII were incorporated at the 5’ end of the forward and reverse primer respectively so that the PCR product of FtsK_C_ could be cloned into a pGEX-KG expression vector via restriction enzyme digestion and ligation. Plasmid containing FtsK_C_ insert was sequenced to confirm correct insertion of PCR product using pGEX reverse primer which yields a sequence covering the FtsK_C_ insert. The resulting sequence was aligned with the expected insert sequence and confirmed to be an exact match (Figure S11).

Sequenced result (1000) TGGTGGAATTCTAGACTCCATGGGAGGACGCCGAAATCGAGCGCGTCGTG

Expected insert (1) -----------------CCATGGGAGGACGCCGAAATCGAGCGCGTCGTG

Sequenced result (1050) AGCGCGATCGCCACCGACACCCCGAACTACGACAGCGAGCTTCAGAACCT

Expected insert (38) AGCGCGATCGCCACCGACACCCCGAACTACGACAGCGAGCTTCAGAACCT

Sequenced result (1100) CAAGACCCGCGACCAGACCGAGTCCGGCGGGGGCGGCGGGGAGATCGGGG

Expected insert (88) CAAGACCCGCGACCAGACCGAGTCCGGCGGGGGCGGCGGGGAGATCGGGG

Sequenced result (1150) AGAAGCTGCGCGAGCGCGACCCGATTTACGAGCAGGCGGTGGAGATCGTC

Expected insert (138) AGAAGCTGCGCGAGCGCGACCCGATTTACGAGCAGGCGGTGGAGATCGTC

Sequenced result (1200) ATCCGCGAGCAGCGGGGCAGCACGTCGCTCCTCCAGCGCGCGCTCGGCAT

Expected insert (188) ATCCGCGAGCAGCGGGGCAGCACGTCGCTCCTCCAGCGCGCGCTCGGCAT

Sequenced result (1250) CGGGTACGGGAAGGCGTCGCGGCTCATCAAAGCCTTCCCC

Expected insert (238) CGGGTACGGGAAGGCGTCGCGGCTCATCAA-GCTTTATA-

**Figure S11.** Alignment of the sequencing result with expected FtsK_C_ insert sequence derived from genome sequence for *Gemmata obscuriglobus* confirming correct that the FtsK_C_ sequence (highlighted in yellow) was inserted into pGEX-KG expression vector.

### Recombinant protein expression and purification

*E. coli* containing FtsK_C_-inserted plasmid was IPTG-induced to overexpress the GST-FtsK_C_ fusion proteins. Total cell extracts of both uninduced and IPTG-induced were visualised on SDS-PAGE gel to confirm induction of the GST-FtsK_C_ fusion protein (Figure S12).


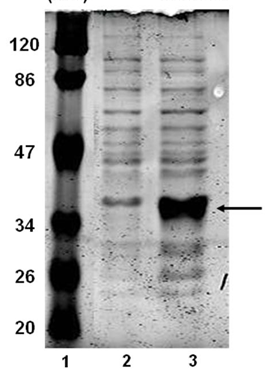


**induced**

**MW**

**Figure S12.** Total cell extracts of *E.coli* BL21transformed with the GST-FtsK_C_ recombinant plasmid. Lane 1 - pre-stained protein MW marker, Lane 2 – total extract from uninduced cells, Lane 3 –total extract from IPTG-induced cells, with clear overproduction of the GST-FtsK_C_ fusion protein ca. 37kDa (arrow).

Total cellular proteins were extracted and GST-FtsKC fusion proteins were captured using affinity chromatography purification with Glutathione Sepharose 4B. Purified fusion proteins were visualised on SDS-PAGE gel and two major bands were observed (Figure S13).


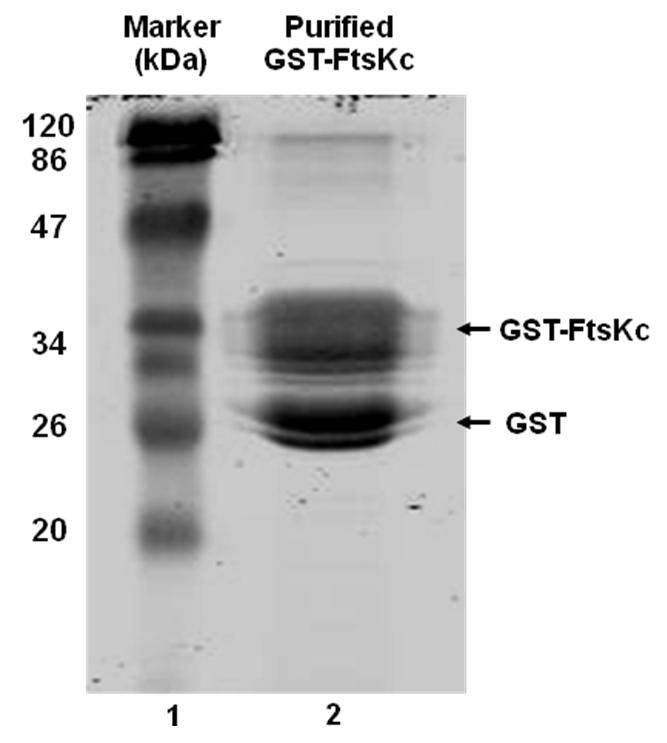


**Figure S13.** GST-FtsK_C_ fusion proteins purified using affinity chromatography and visualised via SDS-PAGE gel. Lane 1 – pre-stained protein MW marker, Lane 2 – purified proteins consisting GST-FtsKC fusion protein and putative GST (arrows).

The upper band in lane 2 represents GST-FtsKC fusion protein with expected molecular weight (37kDa) and the lower major band corresponds to GST with consistent molecular weight (26kDa).

2 ml of purified fusion protein preparation containing 300 µg of protein was sent to IMVS (Institute of Medical and Veterinary Science, Adelaide SA) for polyclonal antibody production in rabbits.

### Testing Anti-FtsK antibody specificity

Anti-FtsK polyclonal antibody raised in rabbit was tested for specificity before immunogold labelling. Test bleed and final bleed-out from rabbit were tested against purified GST-FtsK_C_ fusion protein using ELISA. Figure S14 shows that the both test and final bleed serum bind to GST-FtsK_C_ fusion protein by colour change where negative control pre-immune serum stays colour-less. Table S1 shows the OD_405_ ELISA plate readings demonstrating that the antibody titre for both test bleed and final bleed was 1/800.

Affinity-purified anti-FtsK antibody was then tested for specificity against native FtsK protein in *G. obscuriglobus*. The Western blot result is consistent with a conclusion that purified antibody recognises native FtsK protein in *G. obscuriglobus* cell total extract (Figure S15). There is a clear single band at expected FtsK molecular weight 90 kDa (see arrow).


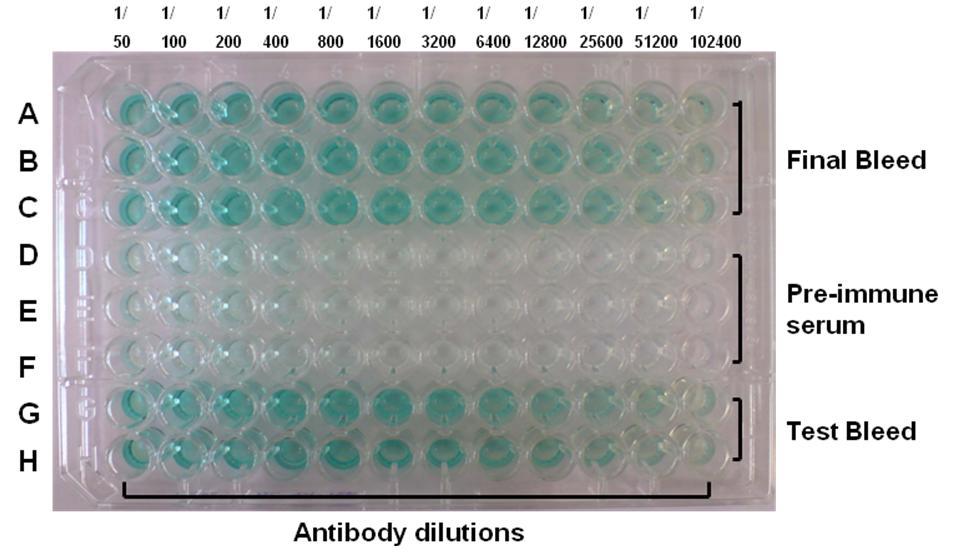


**Figure S14.** ELISA test showing that both the test bleed and final bleed-out specifically recognize purified GST-FtsKc fusion proteins. Green colour change means positive result for both test bleed and final bleed. The negative control (pre-immune serum) shows no colour change.

**Table S1.** OD_405_ readings of the ELISA test assaying the purified GST-FtsK_C_ fusion proteins

| **OD_405_** | **1/50** | **1/100** | **1/200** | **1/400** | **1/800** | **1/1600** | **1/3200** | **1/6400** | **1/12800** | **1/25600** | **1/51200** | **1/102400** |
| --- | --- | --- | --- | --- | --- | --- | --- | --- | --- | --- | --- | --- |
| **A** | 1.564 | 1.66 | 1.668 | 1.63 | 1.672 | 1.625 | 1.495 | 1.365 | 1.178 | 1.018 | 0.822 | 0.638 |
| **B** | 1.64 | 1.655 | 1.663 | 1.662 | 1.677 | 1.617 | 1.505 | 1.389 | 1.242 | 1.041 | 0.842 | 0.65 |
| **C** | 1.651 | 1.64 | 1.686 | 1.679 | 1.695 | 1.603 | 1.466 | 1.318 | 1.163 | 0.986 | 0.773 | 0.597 |
| **Av.** | 1.618 | 1.652 | 1.672 | 1.657 | **1.681** | 1.615 | 1.489 | 1.357 | 1.194 | 1.015 | 0.812 | 0.628 |
| **D** | 0.453 | 0.42 | 0.386 | 0.316 | 0.247 | 0.184 | 0.131 | 0.116 | 0.095 | 0.081 | 0.075 | 0.074 |
| **E** | 0.48 | 0.426 | 0.387 | 0.326 | 0.243 | 0.189 | 0.137 | 0.11 | 0.094 | 0.083 | 0.075 | 0.079 |
| **F** | 0.471 | 0.441 | 0.403 | 0.338 | 0.266 | 0.189 | 0.153 | 0.123 | 0.102 | 0.088 | 0.087 | 0.083 |
| **Av.** | 0.468 | 0.429 | 0.392 | 0.327 | 0.252 | 0.187 | 0.140 | 0.116 | 0.097 | 0.084 | 0.079 | 0.079 |
| **G** | 1.584 | 1.603 | 1.663 | 1.643 | 1.659 | 1.583 | 1.496 | 1.388 | 1.185 | 1.032 | 0.839 | 0.653 |
| **H** | 1.556 | 1.559 | 1.611 | 1.647 | 1.661 | 1.583 | 1.491 | 1.337 | 1.206 | 1.038 | 0.821 | 0.613 |
| **Av.** | 1.57 | 1.581 | 1.637 | 1.645 | **1.66** | 1.583 | 1.494 | 1.363 | 1.196 | 1.035 | 0.83 | 0.633 |

Titre for both test bleed (row G and H) and final bleed-out (row A, B and C) is 1:800 (highlighted in purple).


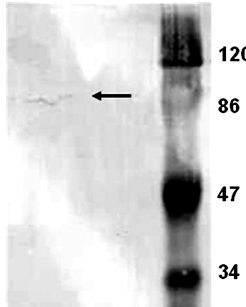


induced

non-

induced

**MW**

**Figure S15.** Western blot results of anti-FtsK probing against extracts obtained from induced and uninduced *G. obscuriglobus* cells. Anti-FtsK antibody at 1:1000 dilution recognizes a protein with expected size (90kDa) for FtsK.

### Immunogold labelling of FtsK in *E.coli* and *G.obscuriglobus*

Affinity-purified anti-FtsK antibody was applied to thin-sections of high-pressure frozen and cryosubstituted *G. obscuriglobus* and *E.coli* cells to detect FtsK distribution. In *G.obscuriglobus* protein A-gold particles indicating antibody labelled several regions including cell nucleoid, nuclear envelope and riboplasm. Figure S16 shows that the nucleoid/nucleoids in each cell was/were heavily labelled by the 10 nm gold particles while riboplasm is less densely labelled. Association of the gold particles with the nucleoid DNA was also observed where the condensed nucleoid appears to be in the process of travelling through the bud neck in a budding cell (Figure S17). The labelling result seems to be specific to a major membrane-bounded compartment, the pirellulosome, since only the pirellulosome (both nuclear body and surrounding riboplasm within intracytoplasmic ICM membrane) compartment of each cell is labelled by gold particles and not the paryphoplasm compartment. This contrast of labelling is illustrated in Figure S18. In some cells the double-membrane nucleoid envelope was also labelled by the gold particles (Figure S19). In contrast to the FtsK location in *G.obscuriglobus*, in *E. coli*, FtsK was found distributed along the periphery of the cell (Figure S20) and is also detected at the division site in dividing cells (Figure S21). In the latter case, the distribution of the labelling suggests that FtsK is associated with a constricting septal ring and its associated membrane as reported previously for *E.coli*.


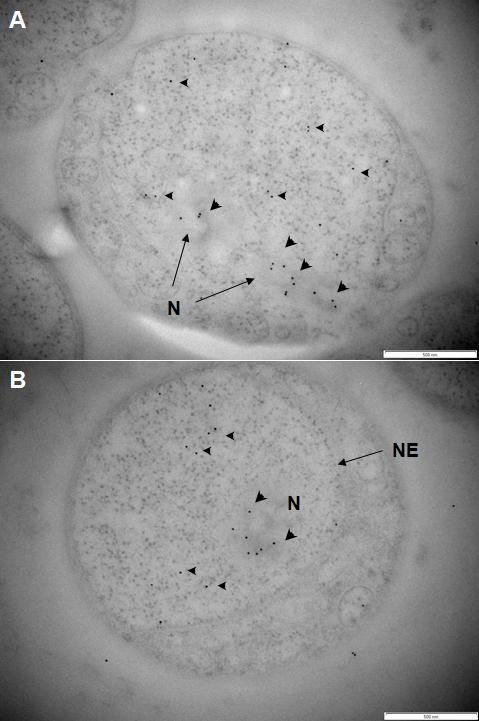


**Figure S16.** Thin-section of high-pressure frozen and cryosubstituted *G. obscuriglobus* cells showing distribution of FtsK using anti-FtsK antibody and protein A-10 nm gold. Both A and B show that the cell nucleoid (**N**) is heavily labelled with gold particles (large arrowheads) and riboplasm is also labelled (small arrowheads) but is less densely labelled compared to the nucleoid. NE – nuclear body envelope. **Bar** – 500 nm.


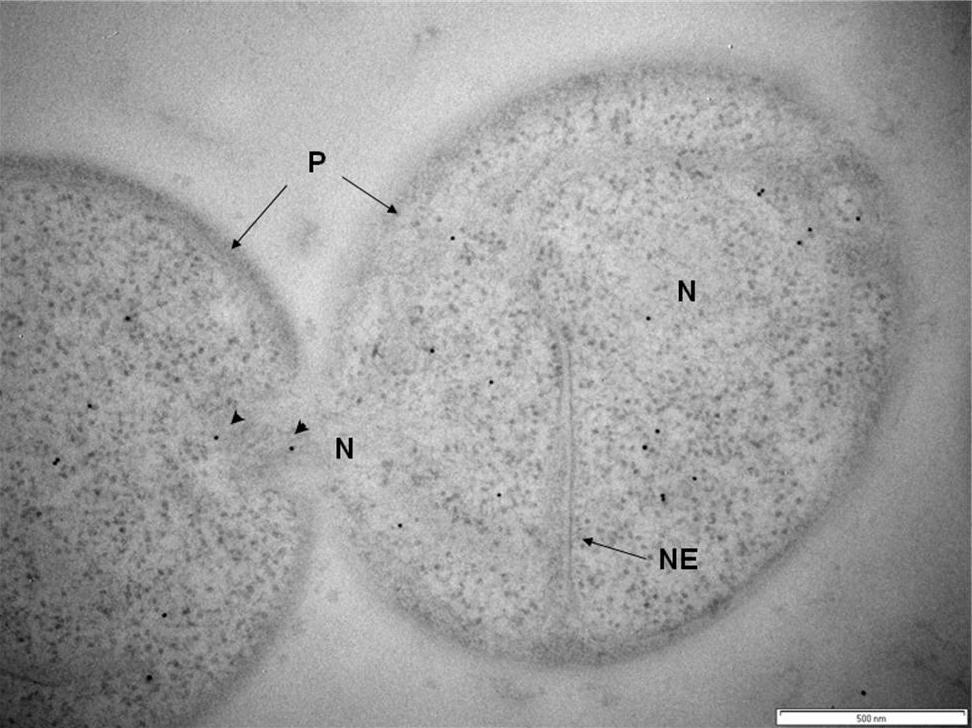


**Figure S17.** Thin-section of high-pressure frozen and cryosubstituted *G. obscuriglobus* cells showing that the cell nucleoid (**N**), while passing through the bud neck, is labelled by the gold particles indicating FtsK (arrowheads). **P** – paryphoplasm, **NE** – nucleoid envelope, **Bar** – 500 nm.


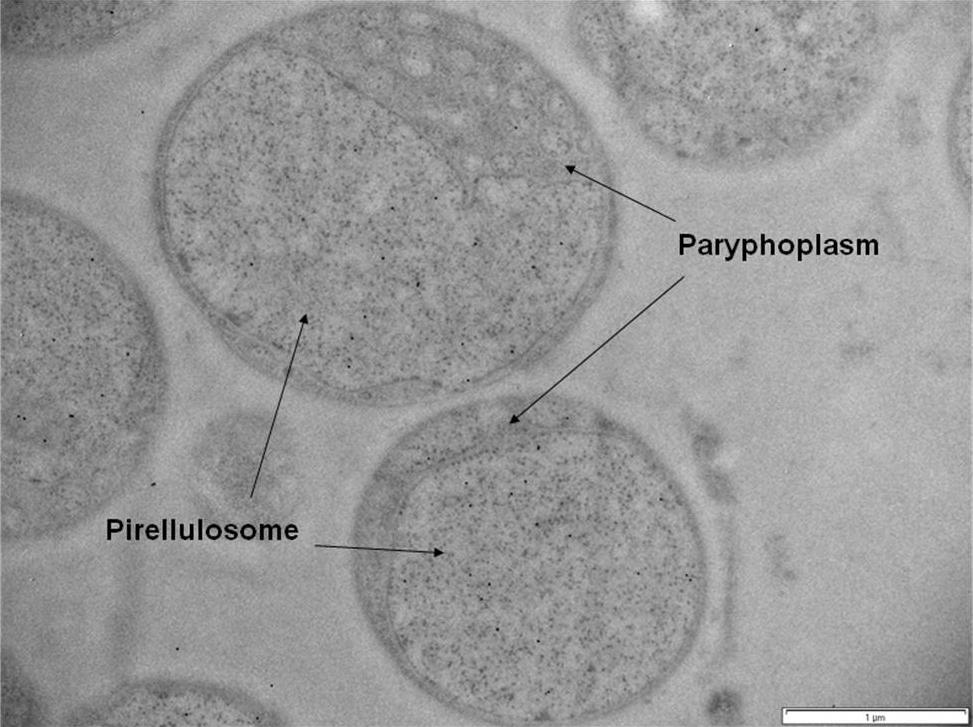


**Figure S18.** Thin-section of high-pressure frozen and cryosubstituted *G. obscuriglobus* cells showing that distribution of FtsK is restricted to the pirellulosome compartment (nuclear body and riboplasm) which displays dense labelling with gold particles compared to the paryphoplasm which has no labelling. **Bar** – 1 µm.


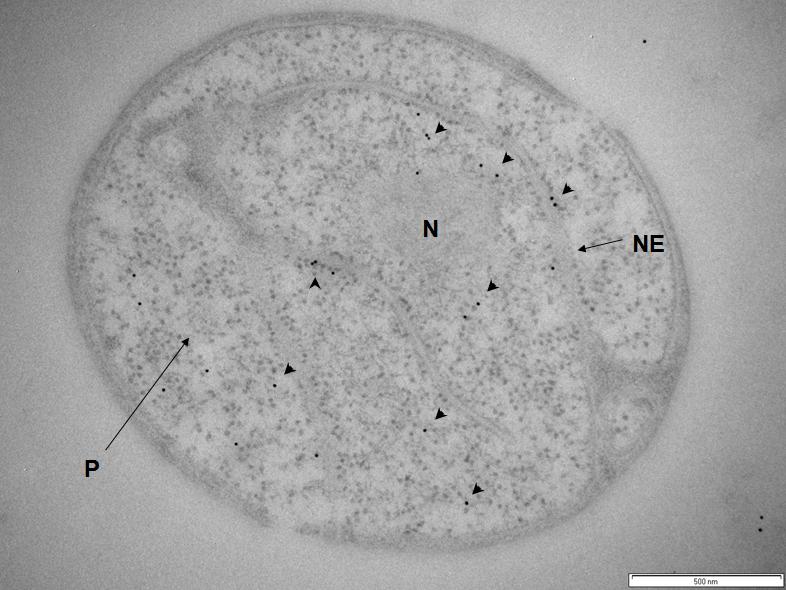


**Figure S19.** Thin-section of high-pressure frozen and cryosubstituted *G. obscuriglobus* cell showing distribution of FtsK, which includes pirellulosome (**P**) and nucleoid envelope (**NE**) indicated by gold particles (arrowheads). **Bar** – 500 nm.


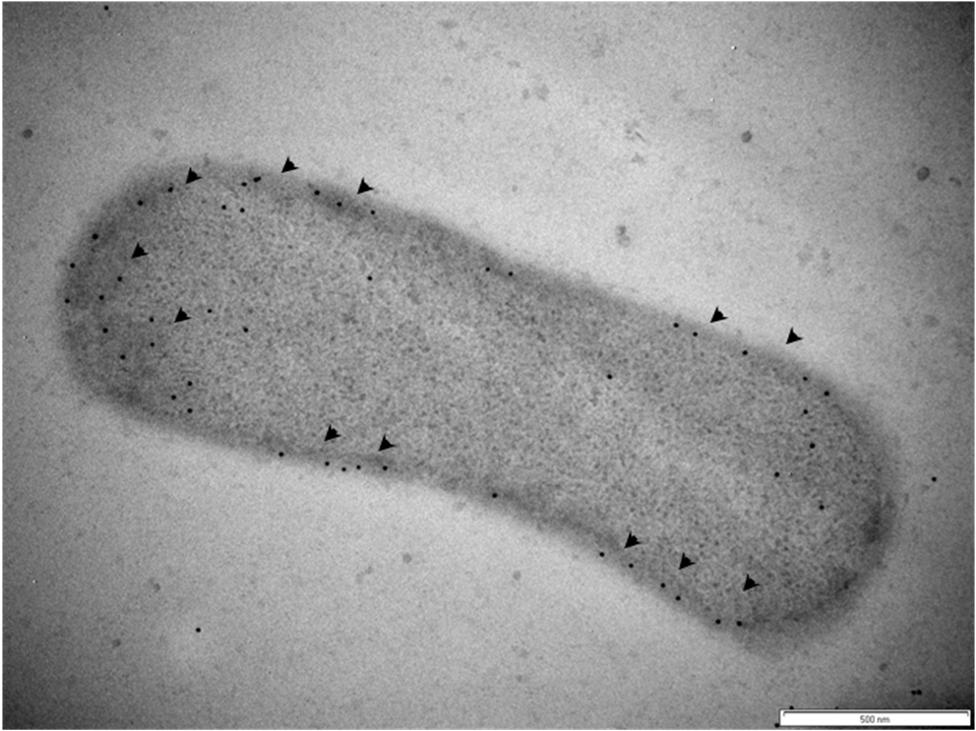


**Figure S20.** Thin-section of a high-pressure frozen and cryosubstituted *E. coli* cell, showing distribution of FtsK along the periphery of the cell (arrowheads). **Bar** – 500 nm.


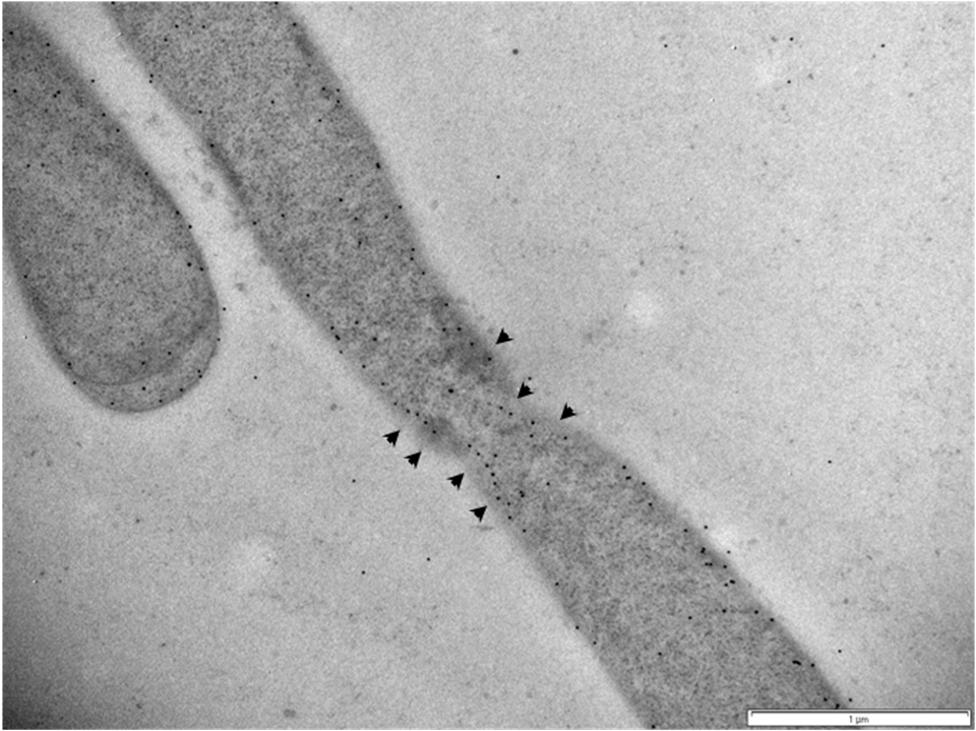


**Figure S21.** Thin-section of high-pressure frozen and cryosubstituted *E. coli* cells, showing distribution of FtsK along the periphery of the cell and at the division site (arrowheads). **Bar** – 1 µm.

FtsK in *G. obscuriglobus* does not seem to be localised within the cell in the same way as in *E. coli*. The cell nucleoid seems to be the most frequently and densely labelled by the gold particles. Table S2 shows the densities of FtsK immunogold labelling in different regions of thin-sectioned *G. obscuriglobus* cells.

**Table S2.** Densities of FtsK immunogold labelling in different regions*

| FtsK localising region | Gold particles/µm^2^ |
| --- | --- |
|  | (mean ± S.E.) |
| Nucleoid | 16.78 ± 1.12 |
| Pirellulosome minus nucleoid | 7.16 ± 0.27 |
| Paryphoplasm | 1.08 ± 0.2 |
| Background | 1.06 ± 0.08 |

* 89 cells were randomly selected and examined for quantitative analysis.

The labelling results were analysed using Student’s t-test with a *P*-value of 0.05 to compare the means of gold particles/µm^2^ of different regions and on this basis there appeared a statistically significant difference between the densities of labelling of the cell nucleoid when compared to other regions of the cell and the background (Figure S22).

**
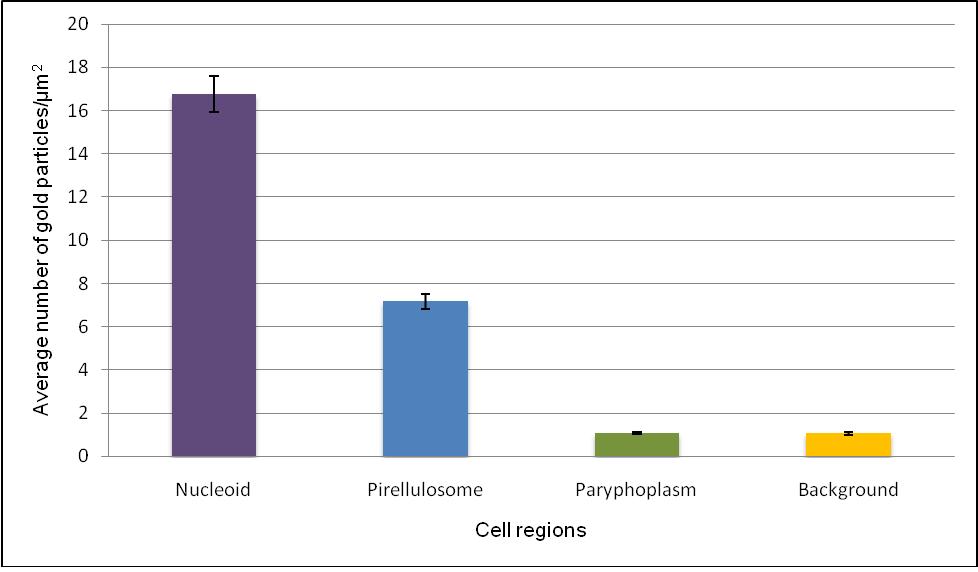
**

**Figure S22.** Average density of gold labelling (gold particles/μm^2^) on *G. obscuriglobus* cells when treated with anti-FtsK antibody. The average labelling density for 3 diferent regions within 89 cells plus the Background labelling density is shown. Error bars shown are the 95% confidence intervals of the mean.

With 16.78 ± 1.12 S.E. gold particles/µm^2^, the nucleoid DNA is twice more densely labelled by the gold particles compared to the rest of the cell cytoplasm i.e. the rest of the pirellulosome or the paryphoplasm. There were no significant differences in the densities of gold labelling between the paryphoplasm and the background, suggesting the low number of gold particles within the paryphoplasm region did not indicate FtsK presence in that region but rather indicated the background labelling level. Association of the gold particles with the nuclear envelope/ICM is much lower compared to the rest of the cell with an average 1 gold particle per cell.
